# Cerebrospinal fluid oligoclonal immunoglobulin gamma bands and long-term disability progression in multiple sclerosis: a retrospective cohort study

**DOI:** 10.1038/s41598-021-94423-x

**Published:** 2021-07-22

**Authors:** Virginija Danylaité Karrenbauer, Sahl Khalid Bedri, Jan Hillert, Ali Manouchehrinia

**Affiliations:** 1 Department of Clinical Neuroscience, Karolinska Institutet, Stockholm, Sweden; 2 Karolinska University Hospital, Tema Neuro, R52, Neurology Medical Unit, 14186 Stockholm, Sweden

**Keywords:** Immunology, Neuroscience, Biomarkers, Health care, Medical research, Neurology, Pathogenesis, Risk factors

## Abstract

Multiple sclerosis (MS) patients with immunoglobulin gamma (IgG) oligoclonal bands (OCB) in the cerebrospinal fluid (CSF) have different genetic backgrounds and brain MRI features compared to those without. In this study, we aimed to determine whether CSF-OCB status is associated with long-term disability outcomes. We used Swedish MS register data on clinically definite MS patients with known OCB status. Date of birth, age at MS onset, and time to sustained Expanded Disability Status Scale (EDSS) milestones 3, 4, and 6; time to conversion to secondary progressive (SP) MS, sex, and immunomodulatory treatment (IMTs) duration were collected. Multivariate Cox regression models were used to investigate the association between OCB status and risk of reaching each milestone. The OCB-positive group reached disability milestones at an earlier time and younger age. OCB-positivity significantly increased the risk of reaching EDSS 3.0 (HR = 1.29, 95% CI 1.12 to 1.48, P < 0.001) and 4.0 (HR = 1.38, 95% CI 1.17 to 1.63, P < 0.001). The OCB-positive group had a 20% higher risk of conversion to SPMS. CSF-OCB presence is associated with higher risk of reaching EDSS milestones and conversion to SPMS. Our findings suggest higher disease modifying effect of OCB presence in the early inflammatory stages of MS**.**

## Introduction

Multiple sclerosis (MS) is an immune-mediated demyelinating and neurodegenerative disorder of the central nervous system (CNS)^1^. Cerebrospinal fluid (CSF) analysis at the time of diagnosis is used for investigation of CSF pathology and for differential diagnosis work up. Routine CSF examination includes measuring oligoclonal bands (OCB) status, IgG index, albumin ratio, and cell counts. OCB in CSF is detected in about 95% of MS patients^2^. Pathological immunoglobulin G (IgG) index predicts OCB positivity^3^. Both CSF-OCBs and CSF-IgG are correlated with adverse MRI, cognitive and disability outcomes both in short and long term observation periods^4,5^. We have previously reported that OCB-negative MS patients have less brain white matter atrophy and less regional gray matter loss in specific areas of the brain including basal ganglia, diencephalon, cerebellum and hippocampus^6^. Results from other studies, however, been inconsistent with regard to clinical and demographic differences such as age at MS onset, sex ratio and disease severity between the two groups^7–10^. Given the paucity of evidence, we conducted a registry based study to investigate whether OCB confers higher risk of long-term disability worsening in MS.

## Results

In total, 7322 patients were included, of whom 828 patients (11.3%) were OCB-negative. Age at MS onset was 1.9 years earlier in OCB-positive group compared with OCB-negative (standardized mean difference (SMD): 0.17, p < 0.001). In the OCB-negative group, platform immunomodulatory treatments (IMTs) (interferons, glatiramer acetate, dimethyl fumarate, teriflunomide) were started on average at a 2.5 years older age in the OCB-negative group compared with OCB-positive (SMD: 0.24, p < 0.001). Similarly, more potent IMTs (natalizumab, alemtuzumab, cladribine, fingolimod, rituximab, autologous stem cell transplantation) were started on average at a 3.2 years older age in the OCB-negative group (SMD: 0.28, p < 0.001). Age at CSF sampling was 2.7 years older in the OCB-negative group (SMD: 0.22, p < 0.001). Table 1 summarized characteristics of the study population.

**Table 1:** Clinicodemographic data of study population, stratified by OCB.

Variables	OCB Negative	OCB Positive	P-value
N	828	6494	
Age at onset (mean (SD))	35.30 (10.77)	33.45 (10.67)	< 0.001
Age at CSF examination (mean (SD))	40.79 (11.99)	38.13 (11.72)	< 0.001
Male (%)	267 (32.6)	1894 (29.2)	0.049
Age at the start of platform IMT (mean (SD))	40.14 (10.47)	37.61 (10.76)	< 0.001
Age at the start of more potent IMT (mean (SD))	42.36 (11.07)	39.24 (10.92)	< 0.001
**Phenotype (%)**			
PP	78 (10.5)	501 (7.9)	
RR	460 (61.8)	4077 (64.1)	
SP	206 (27.7)	1787 (28.1)	
Reached EDSS 3.0 (%)	218 (40.0)	2005 (44.4)	0.055
Reached EDSS 4.0 (%)	177 (27.7)	1511 (29.2)	0.466
Reached EDSS 6.0 (%)	156 (21.7)	1134 (19.9)	0.297
Converted to SP (%)	171 (20.7)	1494 (23.0)	0.139

*SD* standard deviation, *CSF* cerebrospinal fluid, *IMT* disease modifying treatment, *PP* primary progressive, *RR* relapsing remitting, *SP* secondary progressive, *EDSS* expanded disability status scale.

### Age at and risk of sustained EDSS score milestones

**Figure 1:**
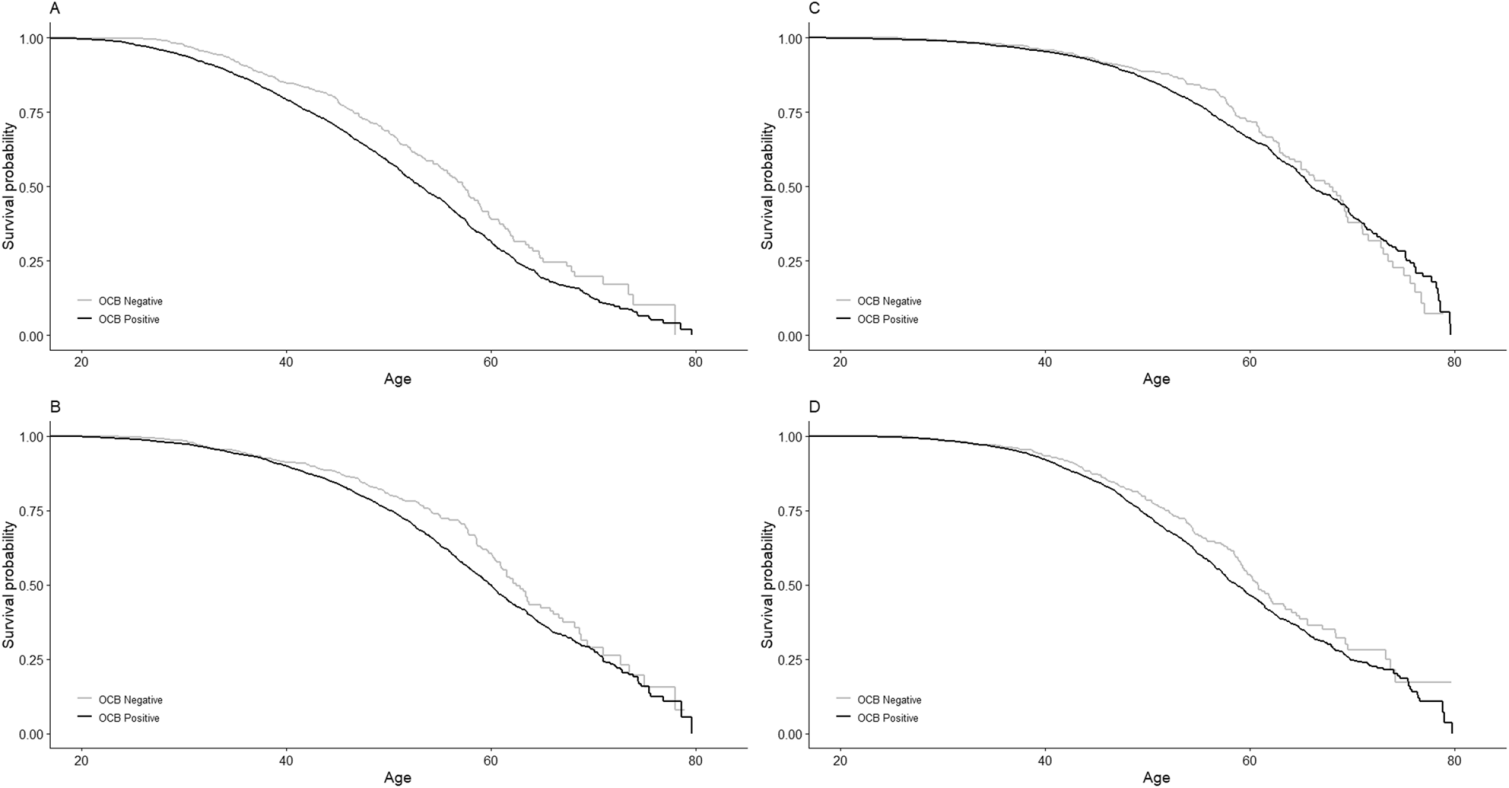
Kaplan–Meier plots (cumulative hazard functions) of reaching EDSS milestones from birth in OCB-positive (black line) and OCB-negative (grey line): (**A**) EDSS 3, (**B**) EDSS 4, (**C**) EDSS 6. (**D**) Kaplan–Meier plot of conversion to SP in OCB-positive (black line) and OCB-negative (grey line).

The median age at reaching sustained EDSS score 3.0 was almost four years younger in OCB-positive patients (57.3 vs. 53.1, Fig. 1A). Similarly, the median ages at reaching sustained EDSS scores 4.0 and 6.0 were almost three (62.5 vs. 60, Fig. 1B) and two (67.8 vs. 66, Fig. 1C) years younger in OCB-positive compared with OCB-negative, respectively. After controlling for potential confounders, OCB-positive patients had a higher risk of reaching sustained EDSS scores 3.0, 4.0 and 6.0 compared to OCB-negative patients, with HRs of 1.29 (95% CI 1.12 to 1.48, P < 0.001, n = 5,055), 1.38 (95% CI 1.17 to 1.63, P < 0.001, n = 5802) and 1.20 (95% CI 0.98 to 1.41, P = 0.08, n = 6,398), respectively.

### Age and risk of conversion to SPMS

After controlling for sex, chronological age, age at MS onset, calendar year of CSF examination and exposure to IMTs (as a time varying covariate), OCB positivity was associated with increased risk of conversion to SPMS (HR: 1.20, 95% CI 1.02 to 1.41, P = 0.03, n = 5,721). Median age at SPMS onset was 58.8 years in the OCB-positive group and 61 years in OCB-negative group (Fig. 1D).

## Discussion

In this study using the national Swedish MS registry data, which covers 80% of prevalent MS patients, we showed that CSF OCB positivity is associated with a higher risk to conversion to the progressive phase of MS and increased risk of reaching EDSS milestones 3 and 4. We observed clinical and demographic differences between OCB-positive and negative patients in agreement with the previously published genetic studies^6,7,11–13^.

In 2013, a meta-analysis regarding the role of OCB in MS and CIS prevalence and prognosis, including 12,253 MS patients from 71 studies, showed that OCB-positive MS patients had a higher risk of reaching disability milestones with an odds ratio of 1.96^14^. Evidence of intrathecal production of immunoglobulins, mostly IgG, has been an important component of MS diagnostic workup^14^. An IgG index greater than 0.7 was found to be associated with OCB positivity and was proposed as an OCB surrogate for MS diagnosis in Asian patients^3^. The prognostic value of OCB and IgG levels have also been studied by others. In a cohort of 38 MS patients, the CSF IgG levels and OCB count at MS diagnosis significantly correlated with EDSS scores after 1 and 5 years, independent of the relapse frequency^4^. Another cohort study of 90 patients showed that the detection of OCB at diagnosis was associated with a shorter time to secondary progression, higher risk of physical and cognitive worsening, and higher load of cortical lesions 10 years after diagnosis^5^. These recent studies are small; our nationwide registry study using a high number of included participants (7,322) supports and confirms that intrathecal IgG synthesis adversely affects MS prognosis. Although OCB is present in 95% of MS patients, thus being highly specific for MS, there has been variable importance placed on the role of CSF OCB in historic MS diagnostic criteria^15–18^. The 2017 revised McDonald criteria included OCB positivity as a means of fulfilling the criterion of dissemination in time in CIS patients with a fulfilled criterion of dissemination in space^18^. Adoption of modern analytical techniques including iso-electric focusing and immunoblotting has greatly increased the proportion of OCB-positive MS cases, leaving only around 10% of patients consistently OCB negative in European MS populations. The findings of genetic and imaging differences between the groups^6,7,11–13^ have prompted a need for renewed assessment of potential clinical differences, as such a distinction would be of importance for how to understand OCB negative MS – as a subgroup of MS or potentially as an entity of its own.

Theoretically, our MS registry cohort might have included a subgroup of myelin oligodendrocyte glycoprotein immunoglobulin G (MOG-IgG) positive patients that are believed to have a more benign disease course. Previous approximations of MOG-IgG seropositivity amongst MS patients has been estimated at 2 in 685 patients, or 0.29%^19^. Based on this, our cohort of 7322 patients may contain approximately 21 MOG-IgG positive patients, of which 18 may be OCB negative if the same OCB proportionality is assumed^20^. This translates to 2% of the OCB-negative patients potentially having MOG seropositivity.

The Swedish MS registry does not record information about MOG serology status.

Our study shows that MS subgroups, separated by OCB status in CSF, develops similar disability progression patterns in time albeit with varying rates of progression as evident on Kaplan–Meier curves. Hence, it is most likely that these two MS subgroups, defined by OCB status in CSF, are very similar and do not create separate disease entities. Our previous publication on familial risk of OCB-positive and OCB-negative groups also indicated that MS lacking OCB is etiologically closely related to the dominant subgroup of OCB positives^21^.

Prognostic value of OCB may be most relevant in early MS where the inflammation is more prominent compared to later stages. It is known that inflammatory events, such as relapses with unfavorable characteristics, and number of gadolinium enhancing lesions early in disease, are associated with adverse long term outcomes^22^, and many studies reported that relapse rate decline with disease duration^23^.

This comprehensive study includes the largest number of OCB negative patients, 828 (11.3%) in total of 7322 MS patients allowing us to calculate a more precise estimation of the prevalence of OCB negative patients in Sweden. Our estimates are twice that of previously reported prevalence by our group (5.5%)^11^ and considered representative of the Swedish MS population, comparable to OCB-negative MS prevalence reported in Scandinavia (12%)^7^. A study on OCB status and association to specific genetic risk alleles in a Scandinavian cohort^7^ also reported a higher prevalence of male gender and older age of onset in OCB negative group.

OCBs are produced by plasma cells which are terminally differentiated B cells. In addition to the presence of OCBs and higher IgG index, the involvement of B cell in MS immunopathology and progression is also supported by the identification of B cell follicles in the meninges of SPMS patients^24,25^. These ectopic B cell follicles can be the site of differentiation of the OCB producing plasma cells^24^. IMTs affect OCB presence in treated MS patients. There has been no reported evidence of effect of rituximab, a monoclonal antibody that primarily target B cells, on OCB presence in CSF^26^, however, conversion to OCB negativity after exposure to natalizumab has been reported in a small subset of patients^27,28^. Histopathological studies had revealed different frequencies of CSF OCB production depending on histopathological MS lesion type: patients with pattern I lesion type harbor OCBs in 88% of cases, whereas patients with pattern II and III lesions had OCB positivity in only 22%. In the latter group, two patients were only transiently OCB-positive^29^. Despite that, OCBs are considered to be a diagnostic mainstay feature in MS^2,30^.

The presence of OCBs indicates inflammation in the CNS^31^, which is believed to precede/lead to neurodegeneration and clinical disability progression^32^; however, their specificity and role in the inflammatory process is still under investigation^31^. In a group of CIS and early MS patients, CSF cell counts were observed to be high in OCB positive patients and correlated with percentage of intrathecal IgG production, in addition the latter correlated strongly with percentage of plasma cells in the CSF^33^. Moreover, the levels of serum neurofilament light chain, a well-established marker for neurodegeneration^34^, is higher in OCB positive patients and correlates with CSF IgG levels^35^, indicating a greater extent of neuro-axonal damage and loss as well as chronic degeneration in OCB positive patients. This is in line with the increased brain atrophy in OCB positive patients but still only confirms the end but not the means or the how.

OCB could be suggested as a potential clinical covariate in randomized clinical trials (RCT). Our current study endpoints were not designed to answer this question, however. In RCT, short term disability progress outcomes are used, such as “confirmed disability progression at 3 months” and “confirmed disability progression” at 6 months^36,37^. International RCT with high number of included RRMS patients (n > 4000) and low EDSS, long time follow up period (3 years) could be characteristics of studies were number of OCB-negative patients would be substantial and time sufficient to evident the OCB effect on disability progression.

Limitations of this study include a high number of patients without recorded OCB status. Hence, Kaplan-Meyer estimates might somewhat be affected by the exclusion of patients that reached milestones but were lacking OCB data. Another limitation of this study is lack of data with regard to factors known to have disease modifying effects such as comorbidities, vitamin D levels, smoking^38–41^ or intrinsic laboratory features (presence of IgM bands in CSF)^42^ and also clinical characteristics^43^, that could potentially alter final results. In addition, one of our study outcomes, conversion to SPMS, lacks standardized objective definition^44^. The definition of SPMS used in this study was “time at which the neurologist has retrospectively evaluated the patients as having at least one year of irreversible disability progression”; this is also a potential source of recruitment bias and is a limitation of the study.

Due to changes in MS diagnostic criteria with time^15–18^, patients fulfilling current criteria were prospectively included in MS registry. That could introduce inclusion bias in to the registry depending on the time period when the patient have been diagnosed with MS and included in registry.

## Conclusion

OCB-positive and OCB-negative MS patients share clinical similarities of long term disability development and conversion to SPMS. However we found that OCB positivity is associated with higher risk of unfavorable outcomes: both reaching disability milestones and SPMS conversion. The effect is small, and more pronounced in early disease stages. Further, our results suggest that OCB status could be considered as a variable in large clinical trials of long term follow up, especially when disability progression is the primary end point.

## Methods

### Study population

We conducted a retrospective registry based study of patients with MS diagnosis according to the McDonald’s diagnostic criteria^15–17^, registered in the Swedish MS registry^45,46^. Patients with available data on OCB status at the time of MS diagnosis, date of birth, date of MS onset, sex, and duration of exposure to first- and second-line immunomodulatory treatment (IMTs) were included. CSF samples that harbored two or more unique CSF bands in comparison to plasma were considered to be OCB positive^2^. Data was extracted on April 2018. Informed consent was obtained from the participants.

### Study outcomes and definitions

Study outcomes included risk of reaching sustained Expanded Disability Status Scale (EDSS) score milestones 3.0, 4.0 and 6.0 and conversion to secondary progressive (SP) MS. Sustained EDSS scores were defined as having reached the milestone of interest, having at least one consecutive EDSS measurement after reaching the outcome and not returning back to a lower EDSS score in any subsequent EDSS measurements. Time at conversion to SPMS was defined as the time at which the practicing neurologist had evaluated MS patient as having at least one retrospective year of irreversible progress of clinical disability^18^.

### Statistical analyses

Clinical and demographic data at the time of CSF examination were compared between OCB-positive and OCB-negative patients using parametric and non-parametric tests for normally and non-normally distributed data, respectively. The Kaplan–Meier method was used to estimate and compare the median age at reaching EDSS milestones and SP conversion between OCB-positive and OCB-negative groups. Multivariate Cox regression models were used to investigate the influence of OCB status on the risk of reaching each clinical milestone. Models were controlled for sex, age at the onset of MS, calendar year of CSF examination, disease phenotype and exposure to IMTs (as time varying covariate). All methods were performed in accordance with the relevant guidelines and regulations**.**

### Ethical approval and consent to participate

Study has been approved by Stockholm´s regional ethical committee on 2017-08-16, ethical permission number 2017/1378-31. Patients included in MS registry give their general consent to use anonymized data for future research.

## Data Availability

The data that support the findings of this study are available from Neuroregister.se but restrictions apply to the availability of this data, which were used under license of Neuroregister Research Board and Ethical permission, issued by Stockholm´s Regional Ethical Committee for the current study, and so are not publicly available. Data are however available from the authors upon reasonable request and with permission of Swedish Neuroregister Research Board, provided that the requesting person holds ethical permission to analyze the data.

## Abbreviations

CI: Confidence interval
CIS: Clinically isolated syndrome
CNS: Central nervous system
CSF: Cerebrospinal fluid
EDSS: Expanded disability status scale
HLA: Human leukocyte antigen
HR: Hazard ratio
IgG: Immunoglobulin G
IgM: Immunoglobulin M
IMT: Immunomodulatory treatment
MS: Multiple sclerosis
OCB: Oligoclonal bands
PP: Primary progressive
RR: Relapsing remitting
SD: Standard deviation
SP: Secondary progressive
SPMS: Secondary progressive multiple sclerosis
